# Japanese Orthopaedic Association Cervical Myelopathy Evaluation Questionnaire (JOACMEQ) in mainland China: an investigation of reliability, validity, and responsiveness

**DOI:** 10.1186/s12955-020-01602-x

**Published:** 2020-10-22

**Authors:** Feifei Zhou, Shuyang Li, Yilong Zhang, Yanbin Zhao, Kevin L. Ju, Fengshan Zhang, Shengfa Pan, Yu Sun

**Affiliations:** 1 Department of Orthopedics, Peking University Third Hospital, Beijing, China; 2 Texas Back Institute Plano, Plano, TX USA

**Keywords:** Japanese orthopaedic association cervical myelopathy evaluation questionnaire, Cervical spondylotic myelopathy, Short form-36, treatment outcome, Post-operative functional recovery

## Abstract

**Background:**

The aim of this study is to investigate the reliability, validity, and responsiveness of JOACMEQ for CSM patients in mainland China.

**Methods:**

A retrospective review was performed on 91 patients with CSM in our hospital from March 2015 to June 2015. Patients completed the JOACMEQ, the mJOA and the SF-36 questionnaires during the process. Cronbach's α was used to evaluate the internal consistency reliability, and test–retest reliability was checked. An exploratory factor analysis was used to determine the correlations among the JOACMEQ questions and the construct validity. The concurrent validity was assessed by Spearman correlation coefficient. The internal responsiveness was determined by effect sizes and standardized response means. External responsiveness was determined by the area under the receiver operating characteristic curve on the basis of the Youden Index.

**Results:**

The mean age of patients was 57.61 years old. The mean follow-up was 24 months. JOACMEQ showed a good internal consistency (Cronbach's α, 0.897). Test–retest reliability showing good result (Pearson's correlation, 0.695–0.905). Our data were amenable to factor analysis (KMO = 0.816, Bartlett's test, χ^2^(45) = 1199.99, p < 0.001), and five factors above 1 were strongly loaded and clustered for each of the five factors. Comparing the scales preoperative to those 2 years postoperative, the average scores of the subscales all increased, and both the ES and SRM showing satisfied responsiveness. In external responsiveness analysis, the recovery rate a appeared to be most responsive to post-operative improvement.

**Conclusions:**

The Simplified Chinese version of JOACMEQ was well-developed with great reliability and sensitive responsiveness. Our study demonstrated that JOACMEQ has content psychometric properties to identify postoperative improvements in CSM patients.

## Background

Cervical spondylotic myelopathy (CSM) is a common cervical degenerative disease in spine surgery [1, 2]. As a clinical syndrome, CSM consists of a pattern of neurologic dysfunction arising from long-term cervical spinal cord compression. Surgical decompression is considered to be the most effective way to prevent disease progression [3–6]. Some patients exhibit improved symptoms after surgery, while some show no changes and still others may exhibit more severe symptoms [7]. Therefore, many researchers have established neurological function rating scales to define the severity of neurological disorders and to evaluate the efficacy of surgical intervention. These scales are called disease-specific outcome measurements. Such scales are only effective for specific diseases or conditions, and are not suitable for other diseases. Of the various outcome measures, the modified Japanese Orthopaedic Association (mJOA) score is one of the most commonly used and established in the literature, which is ranged from – 2 to 17 points. In recent years, evaluations based on the subjective feelings of patients have gradually attracted more attention. These generic outcome measurements have also been widely used with the goal of evaluating patients’ quality of life and have been applied to all types of diseases. A comprehensive clinical assessment should take both disease-specific evaluations and general health evaluations into consideration. We have conducted studies on the assessment of health-related quality of life using the SF-36 in Chinese CSM patients after surgery and its consistency with neurological function assessment [8] and proposed the minimum clinically important difference (MCID) in neurologic function and quality of life after surgery in Chinese CSM patients [9]. To our knowledge, there is currently no evaluation scale that accounts for both disease-specific evaluation and general health evaluation to assess Chinese CSM patients.

Despite the wide acceptance of the mJOA scale worldwide, the authors of the original JOA went on to develop the Japanese Orthopaedic Association Cervical Myelopathy Evaluation Questionnaire (JOACMEQ) in 1999 with an emphasis on assessing patients’ satisfaction, disability, handicaps, and general health from their own perspective, and published in 2007 which was able to assess the status of patients with cervical myelopathy with respect to five functional domains represented by five numerical scores, and could evaluated both outcomes specifically of the disease and of patient’s general health based on patient-reported information. JOACMEQ had been demonstrated to have good validity as well as reliability and correlates strongly with other commonly used neck pain and QOL questionnaires in the Japanese population [10–13]. As far as we know, there is no appropriate mainland Chinese version available for JOACMEQ.

## Methods

### Participants

The study was performed in 103 consecutive patients with CSM treated by the same group of spine surgeons in our hospital from March 2015 to June 2015. All CSM patients were assessed by the Chinese mJOA, the Chinese SF-36 and final Chinese version of JOACMEQ about one week before surgery, a subgroup of 30 patients were each evaluated two times one week apart and all CSM patients were assessed again at around 2 years after surgery. All CSM patients were asked to complete the questionnaires, including the Chinese JOACMEQ, the Chinese mJOA and the Chinese SF-36 during the follow-up.

### Translation

The translation procedure will follow the guidelines described by Beaton et al. [14]. The forward translation was completed by two native Chinese translators. The two translators’ versions and the original version were compared and discussed by the two translators and an orthopaedic professor, until a consensus translation was reached. The back translation was completed by two bilingual translators whose native language was Japanese. They independently translated the Chinese version back into Japanese. Both of these translators lacked a medical background and were not informed or aware of the prior translation procedures. A consensus meeting with all translators was held to compare the back translation with the first Chinese version, original Japanese version, and to resolve discrepancies, ambiguities, or any other problems to reach a preliminary Chinese version of the JOACMEQ. The preliminary version was tested on 10 consecutive patients with CSM to see if any problems arose. All of the translators discussed any issues that were identified, developed the final Chinese version of the JOACMEQ, and performed further psychometric testing.

### Scales

The original JOACMEQ questionnaire included 24 questions, and they were divided into five domains: (1) Cervical Spine Function (4 questions), (2) Upper Extremity Function (3 questions), (3) Lower Extremity Function (5 questions), (4) Bladder Function (4 questions), (5) Quality of Life (8 questions). The mJOA assessment is a method that was released in 1994, which was the revised version of the original Japanese Orthopaedic Association (JOA) scale published in 1975 after several amendments and is used to evaluate spinal cord function of patients with CSM worldwide [15]. The scale comprises 3 parts: motor function part included finger (0–4 points), shoulder & elbow (−2 to 0 points), and lower extremities (0–4 points); sensory function part included upper extremities (0–2 points), trunk (0–2 points) and lower extremities (0–2 points); and bladder function (0–3 points) [21]. The SF-36 is a concise health measurement scale developed by the American Institute of Health in Boston [16]. From a quantitative point of view, this scale provides a more intuitive and comprehensive reflection of the health status of the population. The scale covers the 8 aspects of health-related quality of life: physical function (PF), role-physical (RP), bodily pain (BP), general health (GH), vitality (VT), social function (SF), role-emotional (RE) and mental health (MH). For comparison purposes, the above eight sections can be categorized into the two areas: SF-36 Physical Component Summary (PCS) and SF-36 Mental Component Summary (MCS).

### Psychometric test

Internal consistency reliability was measured by Cronbach’s α: α greater than 0.9 was considered excellent, α of 0.8 or greater was good, α of 0.7 or greater was acceptable, and α less than 0.5 was considered weak [17]. Test–retest reliability was measured by the correlation between the one-week test–retest results. An exploratory factor analysis (principal component analysis with varimax rotation) was carried out to investigate the correlations among the JOACMEQ questions and to compare the factorial structure of the output with those defined by the original JOACMEQ to confirm the construct validity. The concurrent validity (clinical validity) was assessed by calculating the Spearman correlation coefficient to determine the correlation between the JOACMEQ individual domain scores against the SF-36 and mJOA. The internal responsiveness, which attempted to characterize the ability of a scale to detect change over a prespecified time frame, was determined on the basis of effect sizes and standardized response means. General rules for estimating the magnitude of ES or SRM were as follows: < 0.20, trivial effect; 0.20–0.50, small effect; 0.50–0.80, moderate effect; and > 0.80, large effect [18]. In the other words, a higher ES and SRM suggested a better responsiveness. External responsiveness, reflecting the relationship between the change scores against the change in a reference measurement, that was, health transit item from SF-36, was determined by calculating the area under the receiver operating characteristic curve on the basis of the Youden Index to determine the associated sensitivity and specificity.

### Statistical analysis

We used the SPSS19.0 statistical package to establish a database and to conduct data management and analysis. Descriptive statistics including mean, standard deviation (SD), and percentage of the study population were calculated. P value of less than 0.05 was considered significant.

## Results

### Participants

A total of 103 patients completed the preoperative outcome measures. Of these patients, 91 patients completed all of the outcome measures after surgery. The mean age of the patients at baseline was 57.61 ± 10.42 years. Patients were followed for was 24 months after surgery (Table 1).

**Table 1:** Demographics of participants

No. of patients	91	
Age (years, mean ± SD)	57.61 ± 10.42	
Gender	Male:61, Female: 30	
Pathologies of CSM	Cervical disc herniation	58
	OPLL	2
	OPLL with Cervical disc herniation	31

### Reliability

Internal consistency of the questionnaire was analyzed using Cronbach’s α and is shown in Table 2. For upper extremity (0.519) and bladder function (0.568) consistency analysis indicated Cronbach’s α were weak and acceptable. On the other hand, the Cronbach α was acceptable for cervical spine function (α = 0.728), and good for lower extremity function (α = 0.894) and QOL (α = 0.886). When considering the entire 24-question questionnaire as a whole, Cronbach’s α was 0.897, showing a good internal consistency for the scale.

**Table 2:** Internal consistency and test–retest reliability of JOACEMQ subscale

	Item	Cronbach's α		First test	Retest	correlation coefficient
Cervical spine function	4	0.728		79.01 ± 16.35	78.23 ± 16.78	0.758
Upper extremity function	3	0.519		83.49 ± 16.10	81.33 ± 17.23	0.773
Lower extremity function	5	0.894		73.24 ± 14.51	74.32 ± 16.21	0.905
Bladder function	4	0.568		80.48 ± 14.84	80.28 ± 15.67	0.695
Quality of life	8	0.886		50.69 ± 16.39	52.10 ± 15.66	0.796
Total^a^	24	0.897				

^a^We count ‘Total’ for the use of the calculation of Cronbach’s α co-efficient (here ‘Total’ means all categories of JOACMEQ). During the clinical application, we don’t use total score for evaluation, and it’s meaningless to add scores of each item

Test–retest reliability was evaluated for 30 patients enrolled in the study before surgery. The mean score and the Pearson’s correlation of 1st and 2nd visits for each domain were showed in Table 2. Five domains all showed no significant different between the two visits and the Pearson’s correlation were between 0.695 to 0.905, indicating good test–retest reliability.

### Validity

The values from the Kaiser–Meyer–Olkin (KMO) statistic and Bartlett's test of sphericity (KMO = 0.816, Bartlett's test, χ2(45) = 1199.99, p < 0.001) suggested that the data were amenable to factor analysis . During examination of the factor structure of the scale, scale items were released and principal components analysis led to the production of six factors with an eigenvalue above 1. However, when the factor analysis was limited to five, the values shown in Table 3 were obtained. In the rotated component matrix, all of the items were strongly loaded and clustered for each of the five factors (Table 4). While, question 5–2 “Have you been unable to do your work or ordinary activities as well as you would like?” was loaded into both QOL domain and lower extremity function domain.

**Table 3:** Results of factor analysis

Factor	Initial eigenvalues			Rotation sums of squared loadings		
	Eigenvalue	Contribution rate (%)	Cumulative contribution rate (%)	Eigenvalue	Contribution rate (%)	Cumulative contribution rate (%)
1	*7.671*	31.963	31.963	*4.653*	19.387	*19.387*
2	*2.422*	10.091	42.055	*4.522*	18.841	*38.228*
3	*1.923*	8.014	50.069	*2.277*	9.487	*47.715*
4	*1.720*	7.168	57.236	*1.953*	8.136	*55.851*
5	*1.249*	5.205	62.441	*1.582*	6.590	*62.441*
6	*1.080*	4.501	66.942			
7	0.947	3.944	70.886			
8	0.876	3.650	74.536			
9	0.763	3.179	77.715			
10	0.694	2.892	80.607			
11	0.654	2.727	83.334			
12	0.594	2.475	85.809			
13	0.506	2.108	87.917			
14	0.410	1.707	89.624			
15	0.364	1.515	91.139			
16	0.346	1.444	92.582			
17	0.317	1.320	93.902			
18	0.298	1.241	95.143			
19	0.270	1.124	96.267			
20	0.254	1.059	97.326			
21	0.204	0.848	98.174			
22	0.187	0.778	98.952			
23	0.134	0.558	99.509			
24	0.118	0.491	100.000			

Eigenvalues are shown in italics, which represent the total amount of variance that can be explained by a given principal component. In factor analysis, only factors with eigenvalues of 1.00 or higher are traditionally considered worth analyzing, because eigenvalue above 1 means this factor can explain more than 1 variance

**Table 4:** Rotated component matrix for Chinese JOACMEQ

	Factors				
	Quality of life	Lower extremity function	Upper extremity function	Cervical spine function	Bladder function
Q 1–1	0.090	0.052	0.081	*0.761*	0.004
Q 1–2	− 0.113	0.010	0.009	*0.812*	0.169
Q 1–3	0.347	− 0.087	0.310	*0.551*	0.207
Q 1–4	0.258	0.259	0.140	*0.659*	− 0.057
Q 2–1	− 0.020	0.320	*0.758*	0.127	− 0.089
Q 2–2	− 0.019	0.154	*0.869*	0.025	0.196
Q 2–3	− 0.067	0.252	*0.556*	0.399	− 0.066
Q 3–1	0.182	*0.784*	0.181	0.138	0.057
Q 3–2	0.144	*0.646*	0.419	0.063	0.159
Q 3–3	0.186	*0.738*	0.376	0.134	0.142
Q 3–4	0.182	*0.753*	0.240	0.084	0.213
Q 3–5	0.136	*0.754*	0.275	0.074	0.129
Q 4–1	0.205	0.132	− 0.057	− 0.131	*0.505*
Q 4–2	− 0.007	0.445	− 0.338	0.179	*0.538*
Q 4–3	0.024	− 0.061	0.151	0.078	*0.823*
Q 4–4	− 0.051	0.113	0.093	0.155	*0.741*
Q 5–1	*0.577*	0.261	0.025	0.235	0.123
Q 5–2	*0.508*	*0.508*	0.322	0.070	0.167
Q 5–3	*0.649*	0.306	0.172	0.078	0.125
Q 5–4	*0.661*	0.026	0.097	0.283	0.160
Q 5–5	*0.738*	0.038	− 0.123	− 0.021	0.116
Q 5–6	*0.735*	0.155	0.041	0.308	− 0.005
Q 5–7	*0.823*	0.261	0.010	0.169	0.004
Q 5–8	*0.714*	0.202	0.090	− 0.070	0.072

The highest loading of each question in JOACMEQ is shown in italics, which represents this question has the highest correlation with the factors corresponded

Concurrent validity based on the comparison with the SF-36 questionnaire is shown in Table 5. Correlations were found to be good (r = 0.50–75) for qualify of life and moderate for Cervical Spine Function, Upper Extremity Function, Lower Extremity Function, and Bladder Function (r = 0.25–0.50) within the physical component summary (PCS). As for the mental component summary (MCS), correlations were found to be good (r = 0.50–75) for qualify of life, moderate for Cervical Spine Function, Upper Extremity Function, and Bladder Function (r = 0.25–0.50), and poor for Lower Extremity Function (r = 0–0.25).

**Table 5:** Results of concurrent validity

Item	PCS		MCS	
	correlation Coefficient	*P* Value	correlation coefficient	*P* Value
Cervical spine function	0.253	0.009	0.413	< 0.001
Upper extremity function	0.354	< 0.001	0.412	< 0.001
Lower extremity function	0.332	0.001	0.229	0.019
Bladder function	0.472	< 0.001	0.441	< 0.001
Quality of life	0.740	< 0.001	0.729	< 0.001

### Responsiveness

Finally, we evaluated the responsiveness of the JOACMEQ by comparing the scales completed preoperatively to those 2 years after surgery. Relevant data are listed in Table 6. In general, the average scores of the subscales all increased after surgery. The ES and SRM values were ranged from 0.321–1.222, indicating the effects of cervical spine function and bladder function domains were small, the effects of upper extremity function was moderate, the effects of lower extremity and quality of life domains were large, suggesting general responsiveness of JOACMEQ was satisfied.

**Table 6:** Responsiveness for each domain of JOACMEQ

	ES	SRM	AUC
Cervical spine function	0.321	0.432	0.751
Upper extremity function	0.522	0.644	0.693
Lower extremity function	0.812	0.937	0.892
Bladder function	0.342	0.523	0.833
Quality of life	0.884	1.222	0.914

As for the external responsiveness, the ROC curve was used to optimize sensitivity and specificity to distinguish the “Somewhat Better” from the “About the same” patients. The area under the curve (AUC) varied from 0.693 to 0.914, indicating that the ROC curve exhibited suitable accuracy at discriminating between responders and nonresponders. The AUCs for the mJOA score, the mJOA score recovery rate, PCS, and MCS were, 0.892, 0.933, 0.867, and 0.709, respectively. The recovery rate appeared to be the most accurate discriminator of meaningful effectiveness (AUC of 0.933) and appeared to be most responsive to post-operative improvement.

## Discussion

JOACMEQ has been demonstrated to have good validity as well as reliability and correlates strongly with other commonly used neck pain and QOL questionnaires in the Japanese population. Andy Chien [19] (2014) and Cheung PWH [20] (2018) published articles of translation and psychometric testing of JOACMEQ. The results indicate that the Traditional Chinese JOACMEQ successfully retained the psychometric properties of the original JOACMEQ and support the usefulness of the Traditional Chinese JOACMEQ as an appropriate supplementary diagnostic and outcome measure for Taiwan and Hong Kong Chinese patients suspected of cervical spondylotic myelopathy. However, the cultural background and language usage are different in mainland China, and the current versions (Taiwanese Chinese and Hong Kong Chinese versions) were not translated from the original Japanese version, but an English version. Therefore, the aim of this study was to translate the Japanese version of the JOACMEQ into Simplified Chinese and to verify its reliability, validity, and responsiveness in mainland CSM patients.

The translated Simplified Chinese JOACMEQ showed an excellent overall internal consistency, although two domains exhibited weak Cronbach’s α. The internal consistency of the Simplified Chinese version was between that of the Taiwanese Chinese and Hong Kong Chinese versions. As for the test–retest reliability, all domains showed no significant difference between the two visits, and the Pearson’s correlation indicated good test–retest reliability.

In the validity analysis, when the factor analysis was limited to five determinative factors, the five factors explained 62.4% of the variance. The correlation coefficients of all the items with their own domains were significantly higher than the others with the exception of question 5–2. Question 5–2 “Have you been unable to do your work or ordinary activities as well as you would like?” was allocated with both the QOL domain and the lower extremity function domain. This question can be considered as a general health question and can be interpreted very broadly. According to the current data, when patients make assessments of whether their ordinary activities or work are affected by CSM, they are attentive to lower extremity function. For criterion-related validity, besides the QOL domain, results revealed only weak to moderate correlation coefficients when JOACMEQ was compared with SF-36. Cheung PWH (2018) reported that the score of all domains of the translated JOACMEQ had significant correlations (weak to moderate) with all domains of SF-12v2, except for correlations between Lower Extremity Function and Mental Composite Summary, and between Bladder Function and Mental Health. Our data suggested that the QOL domain of the translated JOACMEQ could represent quality of life for CSM patients encompassing both physical and mental components. As for the specific functions affected by CSM, four domains did not show strong correlation with both PCS and MCS of SF-36. Due to this, we consider JOACMEQ to be a better scale for CSM patients than SF-36, as JOACMEQ could cover both disease-specific evaluation and general health evaluation.

As for responsiveness, the responsiveness in three frequently used outcome measurements was explored, examining both internal and external responsiveness. We used two types of effect size (standardized effect size and standardized response mean) and paired t-test to examine internal responsiveness in this study. Results of the current study detected significant changes after the surgical intervention for all domains by the paired t-test. The standardized effect sizes and standardized response means of the JOACMEQ were 0.321–0.884 and 0.432–1.222, respectively. QOL showed the highest ES and SRM, indicating that patients with CSM pay more attention to their QOL improvement than other specific dysfunctions. As our results in internal responsiveness (i.e., moderate to large values of ES and SRM) show, the JOACMEQ appeared useful to assess recovery of CSM patients after surgery. Regarding external responsiveness, area under the curve assessment (AUC) using receiver operating curve is the most common anchor-based method described to evaluate external responsiveness of an outcome instrument. In accordance with the effective size and standardized response mean analysis, the Quality of Life domain was found to have the largest area under the curve. As stated previously, the Chinese JOACMEQ has sufficient ability to detect change when the function of patients improve or deteriorate.

There are several limitations to this study. The main limitation of this study is that this study was carried out at a single center. Secondly, a healthy population was not included in this study. Finally, the anchor utilized in this study for responsiveness is not comprehensive because other anchors, such as the surgeon rating and visual analogue scale (VAS) score, were not included. Further multicenter studies with larger datasets and perhaps longer follow-up times are encouraged to further investigate the psychometric properties of JOACMEQ.

## Conclusions

In summary, the Simplified Chinese version of JOACMEQ was rigorously developed, verified, and compared to other established outcome measures. This study demonstrated that JOACMEQ has satisfactory psychometric properties with good reliability, validity, and sensitive responsiveness to identify postoperative improvements in CSM patients. Thus, we encourage researchers and clinicians to use the Simplified Chinese version JOACMEQ for CSM patients in mainland China.

## Data Availability

The data set supporting the results of this article are included within the article.

## Abbreviations

CSM: Cervical spondylotic myelopathy
mJOA: Modified Japanese Orthopaedic Association
OPLL: Ossification of posterior longitudinal ligament
MCID: Minimum clinically important difference
JOACMEQ: Japanese Orthopaedic Association Cervical Myelopathy Evaluation Questionnaire
JOA: Japanese Orthopaedic Association
PF: Physical function
RP: Role-physical
BP: Bodily pain
GH: General health
VT: Vitality
SF: Social function
RE: Role-emotional
MH: Mental health
PCS: Physical component summary
MCS: Mental component summary
SD: Standard deviation
KMO: Kaiser–Meyer–Olkin
AUC: Area under the curve
VAS: Visual analogue scale
